# Refine gene functional similarity network based on interaction networks

**DOI:** 10.1186/s12859-017-1969-1

**Published:** 2017-12-28

**Authors:** Zhen Tian, Maozu Guo, Chunyu Wang, Xiaoyan Liu, Shiming Wang

**Affiliations:** 1 Department of computer Science and Engineering, Harbin Institute of Technology, Harbin, 150001 People’s Republic of China; 2 School of Electrical and Information Engineering, Beijing University of Civil Engineering and Architecture, Beijing, 100044 People’s Republic of China

**Keywords:** Gene ontology, Topological similarity, Gene functional similarity network, Referenced gene association network

## Abstract

**Background:**

In recent years, biological interaction networks have become the basis of some essential study and achieved success in many applications. Some typical networks such as protein-protein interaction networks have already been investigated systematically. However, little work has been available for the construction of gene functional similarity networks so far. In this research, we will try to build a high reliable gene functional similarity network to promote its further application.

**Results:**

Here, we propose a novel method to construct and refine the gene functional similarity network. It mainly contains three steps. First, we establish an integrated gene functional similarity networks based on different functional similarity calculation methods. Then, we construct a referenced gene-gene association network based on the protein-protein interaction networks. At last, we refine the spurious edges in the integrated gene functional similarity network with the help of the referenced gene-gene association network. Experiment results indicate that the refined gene functional similarity network (RGFSN) exhibits a scale-free, small world and modular architecture, with its degrees fit best to power law distribution. In addition, we conduct protein complex prediction experiment for human based on RGFSN and achieve an outstanding result, which implies it has high reliability and wide application significance.

**Conclusions:**

Our efforts are insightful for constructing and refining gene functional similarity networks, which can be applied to build other high quality biological networks.

**Electronic supplementary material:**

The online version of this article (10.1186/s12859-017-1969-1) contains supplementary material, which is available to authorized users.

## Background

Most cellular components exert their functions through interactions with other cellular components [1]. The development of high-throughput measurement techniques such as tandem affinity purification, two-hybrid assays and mass spectrometry, has produced a large number of data, which is the foundation of biological networks [2]. Biological interaction networks, such protein-protein interaction network, gene regulatory networks, metabolic networks have been well studied and systematically investigated [3]. These networks play important roles in assembling molecular machines through mediating many essential cellular activities [4]. PPI networks occupy a central position in cellular systems biology and provide more opportunities in the exploration of protein functions in various organism [5, 6].

In recent years, some researchers begin to pay their attention to the similarity networks, such as miRNA similarity networks [7–10], gene functional similarity networks [11, 12]. Unlike the traditional interaction networks, similarity networks usually are constructed by measuring the similarity between the nodes in the networks. Since the similarity between each pair of nodes can be measured, these primary similarity networks usually are fully connected. For example, the construction of gene functional similarity networks is by measuring the sequence or ontology similarities between genes. The construction of miRNA functional similarity network is based on the functional similarity of two miRNAs, which can be inferred indirectly by means of their target genes.

However, these fully connected similarity networks have one serious drawback. They do not meet the characteristics of biological network since they are fully connected [13]. Many previous studies have observed that biological networks are generally scale-free and their degree distributions follow the power law or the lognormal distribution [14–16]. From this point of view, we need to prune the unreasonable edges in the fully connected network. In the remainder of this section, we will first review some threshold selection methods, which have applied on gene functional similarity networks and phenotype similarity networks. Then we will put forward the proposed method.

Gene functional similarity networks have been widely used in some fundamental research, such as protein-protein interaction prediction, disease gene identification and cellular localization prediction [11, 17–19]. Rui [11] constructed a gene functional similarity network to infer candidate disease genes on the genomic scale. The gene functional similarity network almost covers twice number of genes in the traditional PPI networks, which can enlarge the search range of candidate genes. However, the constructed gene functional network only keeps 100 nearest neighbors for each gene. As is pointed by Tian [20], this strategy is a very arbitrary for the selection of gene similarity values. Afterwards, Li [17] constructed a corresponding 5-NN network by means of keeping first five nearest neighbors of genes in the fully connected semantic similarity network. This method also has the common shortcomings with method Rui [11]. Besides, Elo [21] put forward a clustering coefficient-based threshold selection method to select a proper threshold for gene expression network. The similarity value below the selected threshold will be set to zero. However, small similarity in biological networks may be meaningful, while large similarity may also be noise. Perkins [22] applied the spectral graph theory on gene co-expression similarity networks for threshold selection. Perkins elaborated that applying a high-pass filter may remove some biologically significant relationships. These methods above always ignore the smaller similarity values, although they are meaningful sometimes.

At the same time, the threshold selection problem for the fully connected networks appears in other type of similarity networks [23–26]. For example, Van [23] made use of text mining method to classify over 5000 human phenotypes in the Online Mendelian Inheritance database and then constructed a fully connected phenotype similarity network. Li [24] employed the phenotype similarity network to infer phenotype-gene relationship. The authors only keep the first five nearest neighbors for each phenotype in the phenotype similarity network and obtain a 5-NN phenotype network. Later, Zhu et al. [25] come up with a new diffusion-based method to prioritize candidate disease genes. They believe that similarity values of phenotypes below the cutoff 0.3 are uninformative. Therefore, they did not considered similarity values below this selected threshold and set them to zero. Zou [27] and Vanunu [26] also keep the edge values higher than 0.3 in the phenotype similarity networks in their experiments. As for the phenotype similarity networks, the threshold selection has the same drawbacks with gene functional similarity network.

Based on the analysis for each method above, we can find that the threshold selection problem for the fully connected network is necessary, which has a significant effect on its applications. To the best of our knowledge, current threshold selection strategies for the fully connected networks are arbitrary or unreasonable. Therefore, it is still a challenge problem that how to construct a reliable gene functional similarity network.

In this article, we proposed a novel method to establish a high quality gene functional similarity network. The contribution of our study is listed as follow.

- We construct an integrated gene functional similarity network based on six different functional similarity calculation methods.
- We built a referenced gene-gene association network based on the PPI networks.
- To the best of our knowledge, this is the first method that tries to refine gene functional similarity network based on a referenced gene-gene association network.

## Methods

**Fig. 1:**
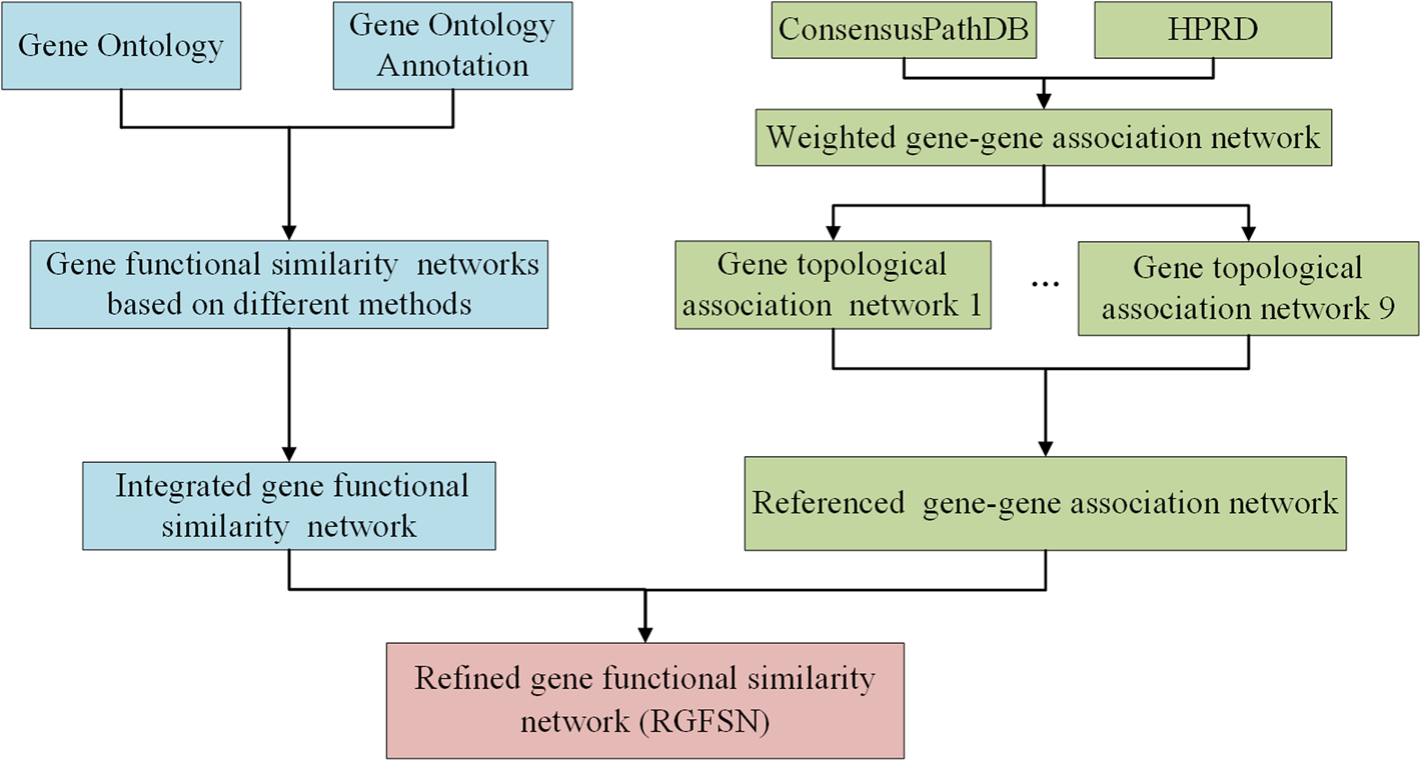
The flowchart for the construction of RGFSN

In this section, we will first introduce the experimental data briefly. Then we construct the integrated gene functional similarity network based on six functional similarity methods. After that, we will employ similarity indices between genes in PPI networks to construct nine gene similarity networks and get the referenced gene-gene association network. In the end, we obtain the refined gene functional similarity with the help of the referenced gene association network. Figure 1 depicts the flowchart of the proposed method.

### Data sources

- Gene Ontology and Gene Ontology Annotation data

We downloaded the Gene Ontology (GO) data from the Gene Ontology database (dated July 2017) which contains 46,929 ontology terms totally subdivided into 4295 cellular components, 30,572 biological process and 12,062 molecular function terms. Gene Ontology Annotations (GOA) data for *H. sapiens* was downloaded from the Gene Ontology database (dated July 2017).

- Protein-protein interaction data

Firstly, we obtain the protein-protein interaction data from human protein reference database (HPRD). HPRD is a high reliable PPI database, which is a resource for experimentally derived information about the human proteome. HPRD totally contains 39,240 interaction relationships relating 9617 proteins. Here, we select the maximum clique of HPRD, which contains 36,900 interaction relationships and 9219 proteins.

ConsensusPathDB are downloaded from the Website (http://consensuspathdb.org/). We selected three typical PPI networks based on ConsensusPathDB [28], which are Reactome, DIP and Biogrid. Specially, Biogrid contains 15,400 genes and 21,468 interactions, while Reactome contains 3332 genes and 19,604 interactions. As for DIP, it contains 3239 genes and 15,964 interactions. In this study, we will construct an integrated referenced gene-gene association network based on the four PPI networks above.

### Construction of integrated gene functional similarity network based on GO and GOA

As we know, GO has three types of ontologies: cellular component (CC), molecular function (MF) and biological process (BP), respectively. Functional similarity between genes can be inferred from the semantic relationships of their annotated GOs [29]. Here we measure gene functional similarity using three types of ontology annotations that contain Inferred Electronic Annotations (IEA).

Since one method may have error prone in measuring functional similarity, the similarity here is calculated by six different kinds of methods. They are Resnik [30], Wang [31], GIC [32], SORA [33], WIS [34] and TopoICSim [35] respectively. Method Resnik, Wang, and TopoICSim are pair-wise approaches, while method GIC, SORA and WIS are group-wise approaches. Besides, with the help of online tools [36, 37], we can measure the gene functional similarity efficiently. In this article, ‘functional similarity’ refers to the similarity between genes, and ‘semantic similarity’ refers to the similarity between two GO terms.

Suppose there are genes *A* and *B*, the functional similarity between genes *A* and *B* can be measured from CC, MF and BP ontologies. Therefore, the functional similarity of gene *A* and *B*is the integration of the three types of functional similarity, which can be measured by Eq. (1).

1 $$ MergedSim\left(A,B\right)=1-\sqrt[3]{\prod \limits_{n=1}^3\left(1-{FunSim}_i\left(A,B\right)\right)} $$

*FunSim*_*n*_(*A*, *B*) (*n* = 1, 2, 3) denotes the functional similarity measure derived from CC, MF and BP similarity, respectively.

As for method Resnik, Wang, GIC, SORA, WIS and TopoICSim, their functional similarity results need to be integrated. The integrated functional similarity between genes *A* and *B* is calculated as follow:

2 $$ Sim\left(A,B\right)=1-\sqrt[6]{\prod \limits_{n=1}^6\left(1-{MergedSim}_n\left(A,B\right)\right)} $$

where *MergedSim*_*n*_(*A*, *B*)(n = 1, 2, 3,4,5,6) denotes the functional similarity method derived from method Resnik, Wang and GIC, SORA, WIS, TopoICSim, respectively.

Applying this operation to all gene pairs, thus we construct the integrated gene functional similarity network. It is noteworthy that the integrated gene functional similarity network is a fully connected network, which we need to purify the spurious edges in it. The number of genes in the integrated gene functional similarity network and PPI network is the same.

### Construction of the referenced gene-gene association network

Here, we will construct a referenced gene-gene association network based on four PPI networks In order to maintain the unity of the number of genes, the genes in Reactome, DIP and Biogrid are the same with that in HPRD. We construct an integrated PPI network based on Reactome, DIP and Biogrid data in ConsensusPathDB and HPRD data. The construction process mainly has three steps.

- Step one: construction of the weighted gene-gene association network

We assess the reliability of protein-protein interactions in the integrated PPI network by edge clustering coefficient (ECC). Edge clustering coefficient is such a measure, which can both evaluate the reliability of interactions in PPI network and describe the association strength of two proteins [38]. For an edge *E*_*x*, *y*_ connecting genes *x* and *y*, the ECC of edge *E*_*x*, *y*_ is defined as.

3 $$ ECC\left(x,y\right)=\frac{z_{x,y}}{\mathit{\min}\left({d}_x-1,{d}_y-1\right)} $$

where *z*_*x*, *y*_ represents the number of triangles that actually include the edge in the network. *d*_*x*_and *d*_*y*_are the degrees of genes *x* and *y*, respectively. *min*(*d*_*x*_ − 1, *d*_*y*_ − 1) denotes the number of triangles that contains the edge *E*_*x*, *y*_ at most. Obviously, the value of *ECC*(*x*, *y*) ranges from 0 to 1. Each pair of protein-coding genes in the integrated PPI network can be measured using Eq. (3), and we can obtain a weighted gene-gene association network.

- Step two: construction of gene topological association networks

For each pair of genes *x* and *y*in weighted gene-gene association network, a similarity score *s*_*xy*_ is assigned to weigh their topological similarity. As we know, a higher similarity score corresponds to a higher probability of forming an association between two genes. Here, we define six similarity indices between two genes in the weighted gene-gene association network, which have been proposed by Yang [39]. They are the Weighted Common Neighbors (WCN), Weighted Resource Allocation (WRA) and Weighted Adamic-Adar (WAA) indices, as well as reliable-route weighted similarity indices [40, 41]. The six similarity indices between genes *x* and *y* are formulated as follows:

(1) Weighted Common Neighbors.

$$ {s}_{xy}^{WCN}=\sum \limits_{z\in {O}_{xy}}{w}_{xz}+{w}_{zy} $$

(2) Weighted Resource Allocation

$$ {s}_{xy}^{WRA}=\sum \limits_{z\in {O}_{xy}}\frac{w_{xz}+{w}_{zy}}{s_z} $$

(3) Weighted Adamic-Adar(WAA)

$$ {s}_{xy}^{WAA}=\sum \limits_{z\in {O}_{xy}}\frac{w_{xz}+{w}_{zy}}{\log \left(1+{s}_z\right)} $$

(4) Reliable-route Weighted Common Neighbors

$$ {s}_{xy}^{rWCN}=\sum \limits_{z\in {O}_{xy}}{w}_{xz}\cdot {w}_{zy} $$

(5) Reliable-route Weighted Resource Allocation

$$ {s}_{xy}^{rWRA}=\sum \limits_{z\in {O}_{xy}}\frac{w_{xz}\cdot {w}_{zy}}{s_z} $$

(6) Reliable-route Weighted Adamic-Ada

$$ {s}_{xy}^{rWAA}=\sum \limits_{z\in {O}_{xy}}\frac{w_{xz}\cdot {w}_{zy}}{\log \left(1+{s}_z\right)} $$

where *O*_*xy*_denotes the common neighbor set of genes *x* and*y*, *w*_*xy*_ represents the weight of the edge linking genes *x* and *y*, *s*_*z*_ denotes the sum of weights for edges linking to*z*.

Then, we will define another three similarity indices. Quasi-local similarity indices [42] not only consider the local similarity of two nodes, but also take local paths between them into account. Therefore, we define weighted reliable local path similarity indices as the similarity metric between unconnected genes *x* and *y*. The weighted reliable local path similarity indices are formulated as follows:

(7) Weighted reliable local path common neighbor index

$$ {s}_{xy}^{rWCNLP}=\sum \limits_{z\in {O}_{xy}}{w}_{xz}\cdot {w}_{zy}+\alpha \sum \limits_{m\in \Gamma (x),n\in \Gamma (y)}{w}_{xm}\cdot {w}_{mn}\cdot {w}_{ny} $$

(8) Weighted reliable local path Resource Allocation index

$$ {s}_{xy}^{rWRALP}=\sum \limits_{z\in {O}_{xy}}\frac{w_{xz}\cdot {w}_{zy}}{s_z}+\alpha \sum \limits_{m\in \Gamma (x),n\in \Gamma (y)}{w}_{xm}\cdot {w}_{mn}\cdot {w}_{ny} $$

(9) Weighted reliable local path Adamic-Adar index

$$ {s}_{xy}^{rWAALP}=\sum \limits_{z\in {O}_{xy}}\frac{w_{xz}\cdot {w}_{zy}}{\log \left(1+{s}_z\right)}+\alpha \sum \limits_{m\in \Gamma (x),n\in \Gamma (y)}{w}_{xm}\cdot {w}_{mn}\cdot {w}_{ny} $$

where Γ(*x*) denotes the neighbor set of gene *x*, *α* is a parameter to adjust the contribution of length-3 paths. In this research, we set *α* as 0.5 to balance the length-3 path.

Applying those nine similarity indices to all gene pairs, we construct nine gene topological association networks, respectively. The edge values in the topological gene association networks denote the topological similarity between gene pairs.

- Step three: construction of the referenced gene-gene association network

By means of integrating the similarity scores in the nine gene topological association networks, we can obtain an integrated gene topological association network, whose edge weight is defined as.

$$ w=\sum \limits_{i=1}^9{\alpha}_i{w}_i $$

where *w*_*i*_ denotes the similarity score of gene pair in the i*th* gene topological association network. *α*_*i*_ is the parameters to weight the nine gene topological association networks. *α*_*i*_ was set as 1/9 to equally weigh the importance of the nine gene topological association networks..

In this article, we call this integrated gene topological association network as the referenced gene-gene association network. The edge values in the referenced gene-gene network denotes the topological similarities between gene pairs. The construction for the referenced gene-gene association network is completed.

### Threshold selection for the integrated gene functional similarity network

Next, we will refine the integrated gene functional similarity network based on the referenced gene-gene association network. For any two genes *A* and *B*, their similarity values in **integrated gene functional similarity network (IGFSN)** and the **referenced gene-gene association network (RGAN)** are represented as *sim*(*A*, *B*)_*IGFSN*_ and *sim*(*A*, *B*)_*RGAN*_, respectively. The similarity value between gene *A* and *B* in the **refined gene functional similarity network (RGFSN)** is denoted as *sim*(*A*, *B*)_*RGFSN*_, which can be calculated by Eq. (4).

4 $$ sim{\left(A,B\right)}_{RGFSN}=\left\{\begin{array}{l} sim{\left(A,B\right)}_{IGFSN}\kern1.5em \mathrm{if}\ \left| sim{\left(A,B\right)}_{IGFSN}- sim\Big(A,B\Big) RGAN\right|<0.1\wedge sim\left(A,B\right) RGAN\ne 0\\ {}\kern8em \\ {}\ 0\kern7em \mathrm{others}\end{array}\right. $$

Applying this operation to all gene pairs in the integrated gene functional similarity network, we can obtain the refined gene functional similarity network (RGFSN). From the Eq. (4), we can find that if the difference of similarity value between genes *A* and *B* in IGFSN and RGAN is large, the similarity value of *A* and *B* in RGFSN will be set to 0. In other words, the similarity value in IGFSN is noise according to RGAN. In this way, we can remove all the spurious edges in IGFSN.

What’s more, taking the depth-first traversal experiment on RGFSN, we find that the refined gene functional similarity network have some isolated genes. The experiments results show that 8501 genes are formed one cluster, while the other genes (264) are isolated from this biggest connected component. As for this type of genes, we decide to add one of their neighbors in the integrated gene functional similarity network, to make RGFSN become one connected graph. At last, we can obtain a connected refined gene functional similarity network called RGFSN.

It is noteworthy that the small similarity value in integrated gene functional similarity network can be reserved based on our proposed method. Comparing with other threshold selection methods which filer out all edges with low similarity values, our method may be more reasonable.

## Results

In this section, we will firstly compare the distributions of functional similarity values of different methods. Then we investigate the relationship between functional similarity values and protein proximity scores. After that, we focus on the global topological properties and the degree distribution of RGFSN. In the end, we conduct protein complex prediction experiment based on RGFSN, for verifying its reliability and application significance.

### The distribution of functional similarity based on different methods

It is well accepted that gene functional similarity calculation methods used in this research have drawbacks [43]. For example, method Resnik has the ‘shallow annotation’ problem, while method Wang fixes the edge value of semantics contributions [31]. As for method GIC, it simply sums up the IC of terms when it measure the IC of a term set. Therefore, we propose a method to integrate the similarity results of the six methods to avoid the shortage of single method.

**Fig. 2:**
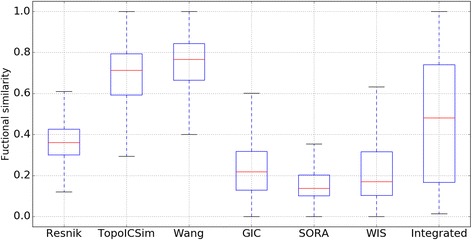
Distribution of functional similarity based on seven different methods. We can find that result for single gene functional similarity method is bias, while the similarity values for the integrated method are distributed from 0 to 1 evenly

We investigate the distribution of six functional similarity methods and the integrated method. We randomly select ten hundred pairs of genes and then measure their functional similarity using method Resnik, TopoICSim Wang, GIC, SORA and WIS. The integrated functional similarity are computed by Eq. (2). The distribution of functional similarity for the four methods are shown in Fig. 2.

From the results, we can clearly find that the highest functional similarity for method Resnik, GIC, WIS and SORA are not lager than 0.65, while the smallest similarity for method Wang is larger than 0.4. Obviously, this does not meet human perspective. By contrast, the integrated results are relatively reasonable. The highest and smallest functional similarity for integrated results are about 1.0 and 0.04, respectively. As a result, it is necessary for us to integrate the results of functional similarity methods.

### Relationship of functional similarity and proximity scores

**Fig. 3:**
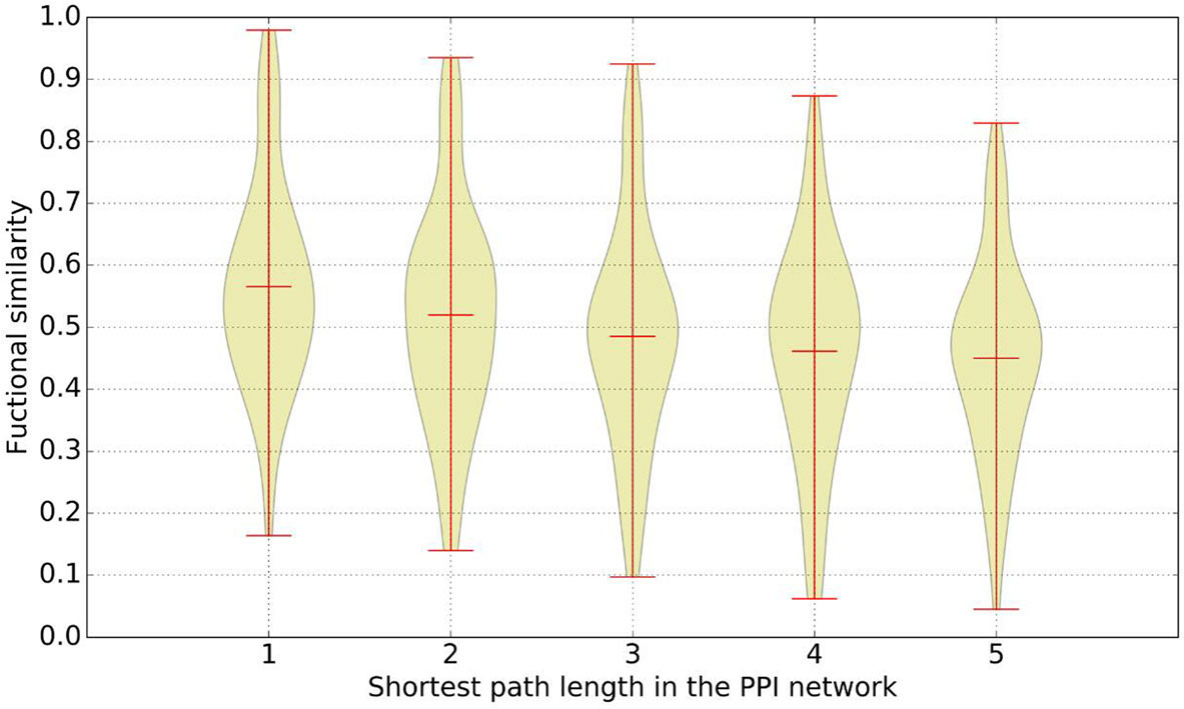
Relationship of gene functional similarity scores and protein proximity scores. Genes with longer path will have smaller functional similarity value

Next, we use the length of the shortest path between two genes in the integrated PPI network as their proximity measure. We choose 100 pairs of genes for each distance (one to five) and measure the functional similarity of gene pairs. To demonstrate the relationship between gene functional similarity scores and protein proximity scores, we draw the violin plot, which are shown in Fig. 3.

From the results, we can clearly find that gene pairs with closer distance (lower proximity scores) will have higher functional similarity scores. For example, the median functional similarity scores for distance one to five are 0.578, 0.519, 0.492, 0.475 and 0.458, respectively. The results indicates that the functional similarity scores are closely consistent with protein proximity scores. Therefore, we can construct a referenced gene-gene association network based on integrated PPI network to refine the gene functional similarity network. From this point of view, the proposed method is reasonable.

### Global topological properties of RGFSN

The biological networks usually have their specific topological characteristics. We analysis the topology attributes of four networks based on Cytoscape 3.4 [44]. The corresponding results are presented in Table 1.

**Table 1:** Summary properties of four biological networks

Property	HPRD	BioGRID	DIP	RGFSN
Number of nodes	9616	20,024	5176	8765
Number of edges	39,239	325,377	22,977	41,646
Cluster coefficient	0.102	0.106	0.098	0.118
Diameter	14	8	10	8
Radius	1	1	1	5
Centralization	0.027	0.102	0.054	0.028
Shortest paths	84,981,088	398,421,606	26,066,196	768,063,238
Characteristic path length	4.209	3.306	3.986	4.158
Average number of neighbors	7.704	23.862	8.742	9.764
Density	0.001	0.001	0.002	0.001
Heterogeneity	1.889	2.347	1.778	1.020

From the results, we can find that the topological properties of RGFSN meet the characteristics of biological networks, which are consistent with three other biological networks. For example, the diameter of a network refers to the longest distance between any two nodes [45]. The diameter of RGFSN is 8, while the diameters for HPRD, BioGRID and DIP networks are 14, 8 and 10, respectively. Besides, the cluster coefficient is a measure of the local interconnectedness of the network, whereas the path length is an indicator of its overall connectedness [46]. For biological networks, the cluster coefficient values are usually in the range 0.1 to 0.5 [47]. The cluster coefficients for HPRD, BioGRID, DIP, RGFSN are 0.102, 0.106, 0.098, and 0.118, respectively. Overall, RGFSN well meets the topological properties of biological networks.

### Degree distribution of RGFSN

**Fig. 4:**
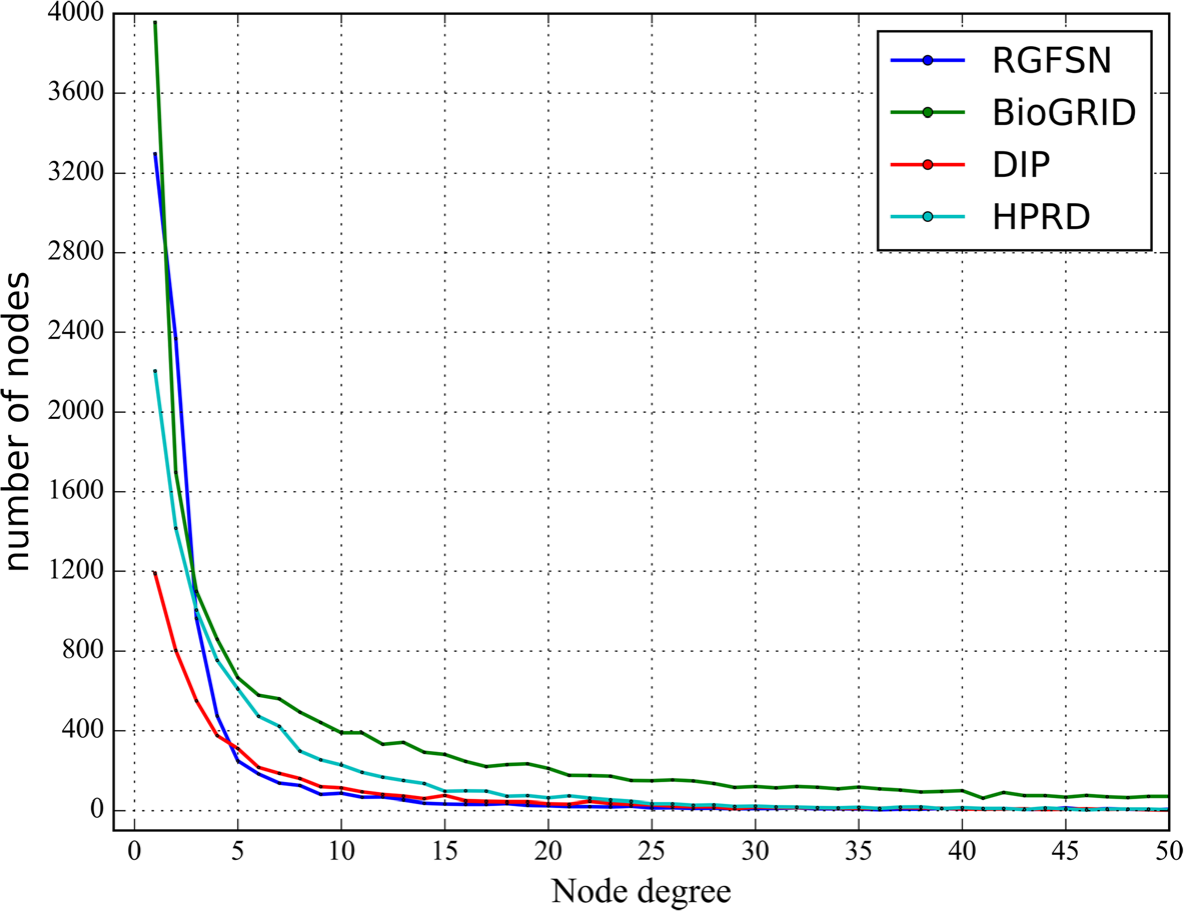
The graphic view of the degree distributions for each network

As is mentioned in previous section, many studies have observed that biological networks are generally scale-free. Their nodal degree distributions usually follow the power law or lognormal distribution [13, 16] [48]. Here we employ four different models to fit the distributions of these four biological networks. These models are Gaussian distribution, power law distribution, log-normal distribution and exponential distribution. All the fitting experiments are conducted on Origin 9. The results are shown in Table 2. Besides, the graphic view of the degree distributions for networks is shown in Fig. 4.

**Table 2:** Four fitting models of degree distribution for each network

Distribution model	P	RGFSN	BioGRID	DIP	HPRD
Gaussian distribution $y={y}_0+\frac{A}{\omega \sqrt{\pi /2}}\exp \left(\frac{-2{\left(x-{x}_c\right)}^2}{\omega}\right)$	*y*_0_	4.26 ± 1.04	2.85 ± 1.09	4.56 ± 0.88	7.03 ± 1.72
	*x*_*c*_	7.80 ± 0.03	1.54 ± 0.06	−8.83 ± 10.13	−0.95 ± 1.12
	*ω*	4.18 ± 0.08	1.51 ± 2.91	3.36 ± 0.18	3.65 ± 2.06
	*A*	7.68 ± 0.07	−5.43 ± 3.12	6.57 ± 1.12	1.02 ± 1.77
	*R* ^2^	0.7652	0.2695	0.9837	0.9822
Power law distribution *y* = *a* ⋅ *x* ^*b*^	*a*	6.64 ± 1.03	3.86 ± 0.035	1.29 ± 0.032	2.38 ± 0.06
	*b*	0.850 ± 0.19	−1.04 ± 0.01	−1.01 ± 0.03	−1.10 ± 0.03
	*R* ^2^	*0.9946*	*0.9945*	0.9628	0.9623
Log-normal distribution $y={y}_0+\frac{A}{\omega x\sqrt{2\pi }}\exp \left(\frac{-{\left(\ln \left(x/{x}_c\right)\right)}^2}{2{\omega}^2}\right)$	*y*_0_	4.89 ± 1.96	3.03 ± 0.94	0.45 ± 4.21	0.84 ± 7.91
	*x*_*c*_	7.36 ± 0.98	1.18 ± 0.26	1.09 ± 0.72	1.09 ± 0.69
	*ω*	0.69 ± 0.10	0.82 ± 0.26	1.12 ± 0.69	1.09 ± 0.67
	*A*	8.17 ± 3.15	5.50 ± 0.44	1.86 ± 0.17	3.45 ± 0.32
	*R* ^2^	0.6469	0.7691	0.6214	0.6205
Exponential distribution *y* = *y*_0_ + *A*_1_ exp(*x*/*t*_1_)	*y*_0_	6.68 ± 1.32	1.30 ± 0.15	1.55 ± 0.25	2.42 ± 5.19
	*A*_1_	9.35 ± 0.96	6.47 ± 0.39	1.58 ± 0.03	2.77 ± 0.06
	*t*_1_	6.68 ± 0.78	1.70 ± 0.11	2.96 ± 0.08	6.35 ± 0.32
	*R* ^2^	0.9816	0.9368	*0.9881*	*0.9853*

The detailed parameters (P) of four fitting models are listed in Table 2. The performances are evaluated by R-squares (R^2^), which provides a measure of how well the data fits a certain model. The results show that RGFSN fits power law distribution best which is followed by exponential distribution. The R^2^ scores for these two models are 0.9946 and 0.9816, respectively. As for BioGRID network, it fits the power law distribution best, while DIP and HPRD networks fit the exponential distribution best. From the results about the degree distributions, we can find that RGFSN has the typical characteristics of biological networks, e.g. scale-free, small world, rather than that of random network.

### Protein complex detection experiment

Protein complexes are groups of associated polypeptide chains whose malfunctions play a vital role. Traditional methods predict protein complexes from protein-protein interaction networks, while some others are based on weighted association networks [43]. Here, we employ CPL [49] algorithm to predict protein complex based on RGFSN.

We verify the effectiveness and rationality of RGFSN by means of assessing the quality of predicted complex. To evaluate the clustering result, we used the jaccard score, which defined as follows:

$$ MatchScore\left(K,R\right)=\frac{\left|{C}_K\cap {C}_R\right|}{\left|{C}_K\cup {C}_R\right|} $$

where K is a predicted cluster and R is a reference complex. Beside, we estimate the cumulative quality of the cluster result and set the *MachScore* as 0.25 [50]. Assume a set of reference complex *R* = {*R*_1_, *R*_2_, *R*_3_, ⋯, *R*_*n*_} and a set of predicted complex *P* = {*P*_1_, *P*_2_, *P*_3_, ⋯, *P*_*m*_}, the recall, the precision and F-measure at complex level are defined as follow.

$$ Rec=\frac{\left|\left\{{R}_i\left|{R}_i\in R\wedge \exists {P}_j\in P,{P}_j{matchR}_i\right.\right\}\right|}{\left|R\right|} $$

$$ Prec=\frac{\left|\left\{{P}_j\left|{P}_j\in P\wedge {R}_i\in R,{R}_i{matchP}_j\right.\right\}\right|}{\left|P\right|} $$

$$ F- measure=\frac{2^{\ast }{Prec}^{\ast } Rec}{Prec+ Rec} $$

A good prediction result should have higher accuracy, recall and F-measure values. The evaluation metrics about the quality of predicted complex have been discussed in detail [50, 51]. In addition, the reference complexes was downloaded from CORUM database [52]. The number of reference complexes for human in this database is 1850 (see Additional file 1).

We construct the 5NN network by keeping five nearest neighbors for each gene in IGFSN, which is proposed by Rui [11]. Here we call this network as the 5NN-IGFSN network. To increase contrast, we conduct the protein complex detection based 5NN-IGFSN with CPL algorithm. Besides, we also conduct protein complex prediction experiment based on HumanNet [53] and STRING [54] networks.

**Fig. 5:**
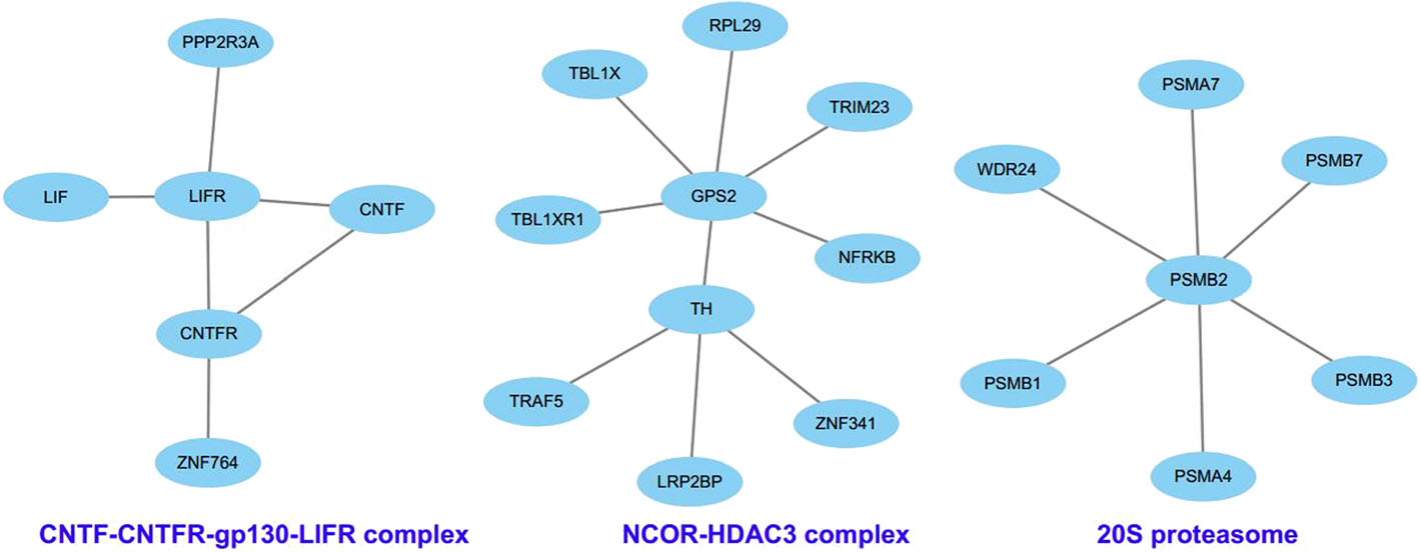
The graph view of three selected predicted protein complex

We evaluate the performance of CPL algorithm on STRING, HumanNet, 5NN-IGFSN and RGFSN according to the evaluation metrics. The results have been shown in Table 3. The precision, recall and F-measure of CPL algorithm based on RGFSN are 0.324, 0.347 and 0.314, respectively, while the results of precision, recall and F-measure for 5NN-IGFSN is 0.275, 0.223 and 0.246, respectively. From this point of view, the best performance in protein complex prediction indicates the reliability of RGFSN. The metric values for STRING and HumanNet are relatively low. The precision, recall and F-measure for STRING is 0.213, 0.268 and 0.236, respectively, while the results for HumanNet is 0.151, 0.142 and 0.146. Since many genes of HumanNet are not in CORUM database, its performance is worst.In the end, we take three examples to demonstrate the predicted results. Three referenced complexes are named as CNTF-CNTFR-gp130-LIFR, NCOR-HDAC3 complex and 20S proteasome, respectively. At the same time, we obtain three predicted complexes based on RGFSN using CPL algorithm. These three predicted complexes are shown in Fig. 5. The high overlap scores between prediction complexes and reference complexes demonstrate that RGFSN is a reliable biological network. The prediction results of CPL on RGFSN are presented (see Additional file 2).

**Table 3:** Results of protein complex prediction based on different networks

Network	Precision	Recall	F-measure
STRING	0.213	0.268	0.236
HumanNet	0.151	0.142	0.146
5NN-IGFSN	0.275	0.223	0.246
RGFSN	0.324	0.347	0.314

## Discussion and conclusions

In this study, we proposed a novel method to construct and refine the gene functional similarity network. Experimental results show that RGFSN is reasonable and effective. Thus, this method can be used to refine gene functional similarity networks effectively. However, two issues need to further study.

### The construction of referenced gene association network

To refine the gene functional similarity network, we have to construct a reliable referenced gene-gene association network. This is the key point for the proposed method. In this study, we construct the PPI network that integrated four PPI data, which are DIP, Biogrid, Reactome and HPRD. The integrated PPI network is reliable and effective.

However, the integrated PPI network has itself shortcomings. It contains about 10,000 genes, which covers less than half of human genes. In addition, the integrated PPI network may be associated with false positives, although it has integrated many PPI networks. Therefore, we have to devote ourselves to seek other proper referenced network to achieve desired results in the next research.

### The verification of the refined gene functional similarity network

How to verify the correctness and rationalization of RGFSN is a very challenging task. This is because there is no direct ways to evaluate the quality of the refined gene functional similarity network. In this research, we verify the rationality and correctness of RGFSN by means of investigating its topological properties and degree distribution. In addition, we predict protein complexes based on RGFSN. The overall experimental results indicate that RGFSN has the typical characteristics of biological networks. We still need to seek other effective methods to validate the rationality of RGFSN in the next study.

## Additional files

Additional file 1: CoreComplexes.xls is the referenced complex downloaded from the CORUM database. (XLS 1637 kb)

Additional file 2: PredictedComplex.xls is the prediction results of CPL algorithm based on RGFSN. (XLS 622 kb)

## References

[1] Barabási A-L, Gulbahce N, Loscalzo J (2011). Network medicine: a network-based approach to human disease. Nat Rev Genet.

[2] Fang Y, Benjamin W, Sun M, Ramani K (2011). Global geometric affinity for revealing high fidelity protein interaction network. PLoS One.

[3] Markowetz F, Spang R (2007). Inferring cellular networks–a review. BMC bioinformatics.

[4] Fang Y, Sun M, Dai G, Ramain K (2016). The intrinsic geometric structure of protein-protein interaction networks for protein interaction prediction. IEEE/ACM Transactions on Computational Biology and Bioinformatics.

[5] Vidal M, Cusick ME, Barabasi A-L (2011). Interactome networks and human disease. Cell.

[6] Zhu L, Deng S-P, Huang D-S (2015). A two-stage geometric method for pruning unreliable links in protein-protein networks. IEEE transactions on nanobioscience.

[7] Wang D, Wang J, Lu M, Song F, Cui Q (2010). Inferring the human microRNA functional similarity and functional network based on microRNA-associated diseases. Bioinformatics.

[8] Luo J, Dai D, Cao B, Yin Y: Inferring human miRNA functional similarity based on gene ontology annotations. In: *Natural Computation, Fuzzy Systems and Knowledge Discovery (ICNC-FSKD), 2016 12th International Conference on:* 2016. IEEE: 1407–1413.

[9] Meng J, Liu D, Luan Y (2015). Inferring plant microRNA functional similarity using a weighted protein-protein interaction network. BMC bioinformatics.

[10] Yu G, Fu G, Wang J, Zhu H (2016). Predicting protein function via semantic integration of multiple networks. IEEE/ACM Transactions on Computational Biology and Bioinformatics.

[11] Jiang R, Gan M, He P (2011). Constructing a gene semantic similarity network for the inference of disease genes. BMC Syst Biol.

[12] Xu Y, Guo M, Liu X, Wang C, Liu Y, Liu G (2016). Identify bilayer modules via pseudo-3D clustering: applications to miRNA-gene bilayer networks. Nucleic Acids Res.

[13] Xu Y, Guo M, Liu X, Wang C, Liu Y (2014). Inferring the soybean (Glycine max) microRNA functional network based on target gene network. Bioinformatics.

[14] Arita M (2005). Scale-freeness and biological networks. J Biochem.

[15] Stumpf MP, Ingram PJ (2005). Probability models for degree distributions of protein interaction networks. EPL (Europhysics Letters).

[16] Khanin R, Wit E (2006). How scale-free are biological networks. J Comput Biol.

[17] Li Y, Li J (2012). Disease gene identification by random walk on multigraphs merging heterogeneous genomic and phenotype data. BMC Genomics.

[18] Schlicker A, Lengauer T, Albrecht M (2010). Improving disease gene prioritization using the semantic similarity of gene ontology terms. Bioinformatics.

[19] Doncheva NT, Kacprowski T, Albrecht M (2012). Recent approaches to the prioritization of candidate disease genes. *Wiley Interdisciplinary Reviews: Systems Biology and*. Medicine.

[20] Tian Z, Guo M, Wang C, Xing L, Wang L, Zhang Y: Constructing an integrated gene similarity network for the identification of disease genes. In: *Bioinformatics and Biomedicine (BIBM), 2016 IEEE International Conference on:* 2016. IEEE: 1663–1668. 10.1186/s13326-017-0141-1 PMC5763299 29297379

[21] Elo LL, Järvenpää H, Orešič M, Lahesmaa R, Aittokallio T (2007). Systematic construction of gene coexpression networks with applications to human T helper cell differentiation process. Bioinformatics.

[22] Perkins AD, Langston MA (2009). Threshold selection in gene co-expression networks using spectral graph theory techniques. BMC bioinformatics.

[23] van Driel MA, Bruggeman J, Vriend G, Brunner HG, Leunissen JA (2006). A text-mining analysis of the human phenome. European journal of human genetics : EJHG.

[24] Li Y, Patra JC (2010). Genome-wide inferring gene-phenotype relationship by walking on the heterogeneous network. Bioinformatics.

[25] Zhu J, Qin Y, Liu T, Wang J, Zheng X (2013). Prioritization of candidate disease genes by topological similarity between disease and protein diffusion profiles. BMC bioinformatics.

[26] Vanunu O, Magger O, Ruppin E, Shlomi T, Sharan R (2010). Associating genes and protein complexes with disease via network propagation. PLoS Comput Biol.

[27] Zeng X, Liao Y, Liu Y, Zou Q (2017). Prediction and validation of disease genes using HeteSim scores. IEEE/ACM Transactions on Computational Biology and Bioinformatics.

[28] Kamburov A, Pentchev K, Galicka H, Wierling C, Lehrach H, Herwig R (2010). ConsensusPathDB: toward a more complete picture of cell biology. Nucleic Acids Res.

[29] Pesquita C, Faria D, Falcao AO, Lord P, Couto FM (2009). Semantic similarity in biomedical ontologies. PLoS Comput Biol.

[30] Resnik P (1999). Semantic similarity in a taxonomy: an information-based measure and its application to problems of ambiguity in natural language. J Artif Intell Res.

[31] Wang JZ, Du Z, Payattakool R, Philip SY, Chen C-F (2007). A new method to measure the semantic similarity of GO terms. Bioinformatics.

[32] Pesquita C, Faria D, Bastos H, Falcão A, Couto F (2007). Evaluating GO-based semantic similarity measures. Proc 10th annual bio-Ontologies meeting.

[33] Teng Z, Guo M, Liu X, Dai Q, Wang C, Xuan P (2013). Measuring gene functional similarity based on group-wise comparison of GO terms. Bioinformatics.

[34] Tian Z, Wang C, Guo M, Liu X, Teng Z (2016). An improved method for functional similarity analysis of genes based on gene ontology. BMC Syst Biol.

[35] Ehsani R, Drablos F (2016). TopoICSim: a new semantic similarity measure based on gene ontology. BMC bioinformatics.

[36] Tian Z, Wang C, Guo M, Liu X, Teng Z (2016). SGFSC: speeding the gene functional similarity calculation based on hash tables. BMC bioinformatics.

[37] Peng J, Li H, Liu Y, Juan L, Jiang Q, Wang Y, Chen J (2016). InteGO2: a web tool for measuring and visualizing gene semantic similarities using gene ontology. BMC Genomics.

[38] Wang J, Li M, Wang H, Pan Y (2012). Identification of essential proteins based on edge clustering coefficient. IEEE/ACM Transactions on Computational Biology and Bioinformatics.

[39] Yang J, Yang T, Wu D, Lin L, Yang F, Zhao J (2017). The integration of weighted human gene association networks based on link prediction. BMC Syst Biol.

[40] Zhao J, Miao L, Yang J, Fang H, Zhang Q-M, Nie M, Holme P, Zhou T (2015). Prediction of links and weights in networks by reliable routes. Sci Rep.

[41] Lü L, Zhou T (2010). Link prediction in weighted networks: the role of weak ties. EPL (Europhysics Letters).

[42] Meng B, Ke H, Yi T (2011). Link prediction based on a semi-local similarity index. *Chinese*. Phys B.

[43] Mazandu G K, Chimusa E R, Mulder N J. Gene Ontology semantic similarity tools: survey on features and challenges for biological knowledge discovery[J]. Briefings in Bioinformatics, 2016:1-16. 10.1093/bib/bbw067 27473066

[44] Assenov Y, Ramírez F, Schelhorn S-E, Lengauer T, Albrecht M (2008). Computing topological parameters of biological networks. Bioinformatics.

[45] Moskvina A, Liu J: How to build your network? a structural analysis. *arXiv preprint arXiv:*160503644 2016.

[46] Stam C, Jones B, Nolte G, Breakspear M, Scheltens P (2007). Small-world networks and functional connectivity in Alzheimer's disease. Cereb Cortex.

[47] Girvan M, Newman ME (2002). Community structure in social and biological networks. Proc Natl Acad Sci.

[48] Pržulj N, Corneil DG, Jurisica I (2004). Modeling interactome: scale-free or geometric?. Bioinformatics.

[49] Dai Q-G, Guo M-Z, Liu X-Y, Teng Z-X, Wang C-Y (2014). CPL: detecting protein complexes by propagating labels on protein-protein interaction network. J Comput Sci Technol.

[50] Zaki N, Efimov D, Berengueres J (2013). Protein complex detection using interaction reliability assessment and weighted clustering coefficient. BMC bioinformatics.

[51] Ramadan E, Naef A, Ahmed M (2016). Protein complexes predictions within protein interaction networks using genetic algorithms. BMC bioinformatics.

[52] Ruepp A, Waegele B, Lechner M, Brauner B, Dunger-Kaltenbach I, Fobo G, Frishman G, Montrone C, Mewes H-W (2010). CORUM: the comprehensive resource of mammalian protein complexes—2009. Nucleic Acids Res.

[53] Lee I, Blom UM, Wang PI, Shim JE, Marcotte EM (2011). Prioritizing candidate disease genes by network-based boosting of genome-wide association data. Genome Res.

[54] Szklarczyk D, Franceschini A, Wyder S, Forslund K, Heller D, Huerta-Cepas J, Simonovic M, Roth A, Santos A, Tsafou KP (2014). STRING v10: protein–protein interaction networks, integrated over the tree of life. Nucleic Acids Res.

